# The evolutionary and coevolutionary consequences of defensive microbes for host-parasite interactions

**DOI:** 10.1186/s12862-017-1030-z

**Published:** 2017-08-14

**Authors:** Kayla C. King, Michael B. Bonsall

**Affiliations:** Department of Zoology, University of Oxford, South Parks Road, Oxford, OX1 3PS UK

**Keywords:** Defensive symbiosis, Host-parasite coevolution, Life-history modelling, Fitness set, Theoretical evolutionary biology

## Abstract

**Background:**

Animal and plant species can harbour microbes that provide them with protection against enemies. These beneficial microbes can be a significant component of host defence that complement or replaces a repertoire of immunity, but they can also be costly. Given their impact on host and parasite fitness, *defensive microbes* have the potential to influence host-parasite interactions on an evolutionary timescale.

**Results:**

Using a phenotypic framework, we explore the evolutionary and coevolutionary dynamics of a host-parasite interaction in the presence of defensive microbes. We show that costs of host-defensive microbe systems are critical in determining whether a defensive microbe based system or an immune system provides better host protection investment. Partitioning the coevolutionary dynamics yields testable predictions. The density of defensive microbes influences the strength of selection resulting from host - defensive microbe - parasite coevolutionary interactions. We find that they lessen the negative effects of infection on hosts and reduce infectivity by directly competing with parasites.

**Conclusions:**

Defensive microbes might thus play a central role in host-parasite interactions, by outright replacing host-based defences, engaging in within-host competition with parasites, and ultimately driving tripartite coevolutionary dynamics.

**Electronic supplementary material:**

The online version of this article (doi:10.1186/s12862-017-1030-z) contains supplementary material, which is available to authorized users.

## Background

Microbes living on or within organisms can have significant effects on host biology [1–4]. Widespread among natural populations of animals [3, 5–7], plants [8, 9] and even humans [10] is the phenomenon known as ‘defensive symbiosis’ whereby microbes protect their hosts against enemy infection [11], whether by parasites or parasitoids. Microbes can reduce parasite infectivity by competing directly via toxin production or indirectly via resource extraction and host-mediated processes [12]. Several classic examples of defensive symbiosis exist. The inherited bacteria *Hamiltonella* in aphids produces toxins which directly kill developing wasp parasitoid larvae [13], and in grasses, fungal endophytes produce alkaloids in grass host tissue making them toxic to herbivores [14]. Possessing these defensive microbes can be costly [15, 16]. Nevertheless, this form of protection provided by microbes can be a significant component of host defence [17–19].

By directly impacting the fitness of hosts and their parasites, these beneficial microbes have the potential to shape their evolution. For instance, it has been shown, theoretically, that defensive microbes (e.g. *Wolbachia*) could diminish the selective effects imposed by parasite infection by altering host life history traits [20]. To what extent the evolution of host-based resistance strategies to coevolving parasites are affected by this additional line of defence remains to be thoroughly empirically tested. Although, the selective effects of defensive microbes on parasite infectivity and virulence are only now being explored [21, 22] the presence of parasites has been shown to facilitate the establishment of defensive microbes in a host population [23] and drive the adaptive origin of host-protective traits in microbes [24]. That defensive microbes can underlie host variation in host resistance and parasite infectivity in some systems suggests host-parasite coevolutionary interactions can be affected. Whole microbial communities and individual microbial symbiont species are documented to be associated with specific host genotypes driving patterns of host-parasite specificity [25, 26]. A degree of interaction specificity is a fundamental assumption of coevolutionary dynamics such as gene-for-gene or matching allele models [27]. If parasites adapt to these genotype-specific microbes [25, 28] then defensive microbes certainly have the potential to mediate host-parasite coevolutionary interactions [29] and even coevolve with the host and/or parasite.

Here, we use a phenotypic framework (e.g., [30–35]) to develop insights into the evolutionary and coevolutionary dynamics of host and parasites in the presence of (costly) defensive microbes. In particular, we explore: (i) the conditions under which hosts should invest in innate immunity or acquire immunity through defensive microbes, and (ii) the consequences of defensive microbes for tripartite coevolution between hosts, defensive microbes, and parasites. Under our set of conditions, the strategy associated with using defensive microbes can invade and spread throughout an otherwise immune system-defended host population. Moreover, we find that defensive microbes can offset their energetic and metabolic costs to the host when parasites are highly abundant. By competing with parasites within the host and reducing their infectivity, defensive microbes can be the central drivers of tripartite coevolutionary interactions.

## Methods

### Mathematical framework

**Fig. 1:**
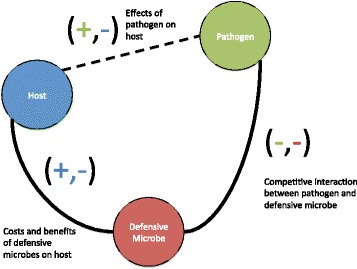
Schematic of host (*blue*) - pathogen (*green*) - defensive microbe (*red*) interaction. The host is negatively affected by the pathogen while the pathogen is positively affected by the host. Pathogen and defensive microbes compete (negative-negative interactions). Defensive microbe has both positive (as it affords protection) and negative (as it imposes fecundity costs) effects on host. The evolutionary and coevolutionary dynamics depend on the fitness sets associated with pairwise and full three-species interactions

We begin by describing the full dynamics of the system (see Fig. 1) in which we build on existing epidemiological frameworks where hosts acquire infections from a free-living parasite (e.g. [36]). This pathogen kills its host and is representative of many invertebrate infections where the parasite induces host mortality. Similar approaches have been widely used to investigate the range of non-linear dynamics that parasites can generate in host populations (e.g. [36–38]). However, the principal aim of our work here is to investigate the population and evolutionary demography of the host and the parasite in presence of a defensive microbe (*D*_*m*_). Defensive microbes are only found within hosts (and are assumed, implicitly, to be vertically acquired by hosts). Moreover, as the interaction between the parasite and the defensive microbe operate at a different temporal scale from the epidemiological dynamics of host infections, this necessitates separating the within-host dynamics for the defensive microbe and the parasite from the between-host epidemiological processes. In doing this we allow within-host process such as competitive suppression and reduced replication of the parasite by the defensive microbe to act independently from the between-host epidemiological process of transmission. We begin by describing the within-host dynamics and then develop an appropriate approach for scaling these within-host and the between-host temporal processes.

#### Within-host processes

Within a host, parasites and defensive microbes are expected to interact. We define the temporal scale of this interaction as *Δ* *T*. The parasite (*P*) dynamics are then described by:

1 $$ \frac{\Delta P}{\Delta T}=\left[\overbrace{\gamma(D_{m})}^{replication}-\overbrace{\beta_{1} D_{m}}^{competition} -\overbrace{\mu_{P}}^{death}\right]P  $$

where *γ*(*D*_*m*_) is the replication rate of the parasite in the presence of the defensive microbe (*γ*(*D*_*m*_)=*γ*_*max*_/(1+*D*_*m*_)), *γ*_*max*_ is the maximum parasite replication rate, *β*_1_ is the strength of competition between the parasite and the defensive microbe and *μ*_*P*_ is the parasite death rate. Direct competition between defensive microbes and parasites is the most commonly observed mechanism underlying microbe-mediated protection in natural systems [39].

Defensive microbes are able to accumulate within the host, and thus benefit from their interaction with the host. Defensive microbes (*D*_*m*_) dynamics are described by:

2 $$ \frac{\Delta D_{m}}{\Delta T}=\left[\overbrace{-\beta_{2} P}^{competition} -\overbrace{\mu_{Dm}}^{death} + \overbrace{h(D_{m})}^{growth}\right]D_{m}  $$

where *β*_2_ is the strength of competition between the defensive microbe and the parasite, *μ*_*Dm*_ is the death rate of the defensive microbe and *h*(*D*_*m*_) is the replication (growth) rate of defensive microbes in the host which follows a logistic growth function *h*(*D*_*m*_)=*r*_*Dm*_(1−*D*_*m*_/*K*_*Dm*_).

To scale the dynamics of these within-host processes to the epidemiological level we assume that the between-host processes operate on a temporal scale *t* and there are *n* iterations of these within-host process for each change in the between-host (epidemiological) dynamics. We can then define *Δ* *T* *n*=*t*. As the derivative (for parasite density Eq. 1) ($\frac {\Delta P}{\Delta T}=P'(t-\Delta T)$) can be written as a finite-difference expression:

3 $$  P'(t-\Delta T)=\frac{\Delta P}{\Delta T}=\frac{P(t)-P(t-\Delta T)}{\Delta T}  $$

then by rearranging this expression (Eq. 3) and substituting in the between-host temporal scaling yields:

4 $$  P(t)\approx P\left(t-\frac{t}{n}\right)+P'\left(t-\frac{t}{n}\right)\left(\frac{t}{n}\right).  $$

The density of the parasite population at time *t*, *P*(*t*) is then the density of the parasites $t-\frac {t}{n}$ steps ago, $P(t-\frac {t}{n})$ plus the change in the parasite density, $P'(t-\frac {t}{n})(\frac {t}{n})$ over the small time interval. We use this small time delay approximation to determine the parasite density (and using a similar expression for the defensive microbe density) from the within-host processes.

#### Between-host processes

The epidemiological processes of infection are described by the interaction between the host and the free-living parasite. Host (*H*) dynamics are then described by:

5 $$ {{\begin{aligned} \frac{dH}{dt}=\left[\overbrace{f(r,D_{m})}^{births}-\overbrace{\lambda(D_{m}) \int_{0}^{\infty} \psi(x) P(x)}^{infection}dx -\overbrace{g(\mu,D_{m})}^{deaths}\right] H \end{aligned}}}  $$

where *f*(*r,D*_*m*_) is the host birth rate and *g*(*μ*,*D*_*m*_) is the host death rate, both of which may be influenced by (costly) microbes (*D*_*m*_). $\int _{0}^{\infty } \psi (x) P(x) dx$ is the expected parasite density in the environment (e.g., [36]), where *P*(*x*) is the probability density of parasites and *ψ*(*x*) is the number of parasites per host. Defensive microbe densities change at the within-host scale, and epidemiological processes are assumed not to affect defensive microbes at the between-host scale. *λ*(*D*_*m*_) is a function for the parasite infection rate (which is reduced as defensive microbe density increases). A simple function for this is:

6 $$  \lambda(D_{m})=\frac{\lambda_{max}}{1+D_{m}}  $$

where *λ*_*max*_ is the maximum transmission rate of the parasite which decreases non-linearly as defensive microbe density (*D*_*m*_) increases.

#### Analysis

Analysis of the model proceeds in a number of ways. First, we begin by determining fitness and the strength of selection on the host and parasite in the presence of defensive microbes (Eqs. 1-4). Second, we determine the evolutionary invasion dynamics of different host defence strategies under costs and benefits of defensive microbes. As noted above, we explore hypotheses that hosts that invest in innate immunity have different evolutionary consequences than hosts that acquire immunity through defensive microbes. Finally, we explore the coevolutionary dynamics of hosts and parasites in the presence of (costly and competitive) defensive microbes.

## Results

### Host fitness and strength of selection

**Fig. 2:**
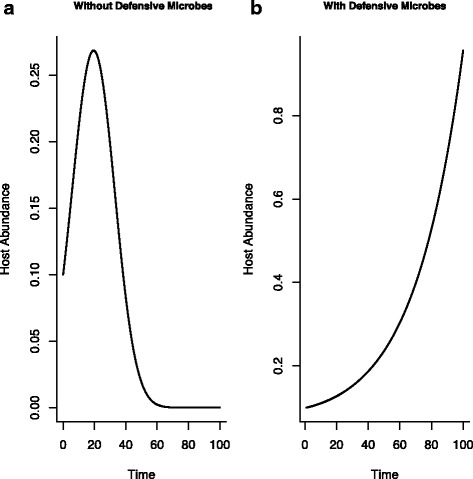
Host dynamics in the **a** absence and **b** presence of defensive microbes. Without defensive microbes, the parasite negatively affects host dynamics albeit in a time-lagged way (mediated through the scaling equation (Eq. 3)). Defensive microbe based immunity can reduce the parasite infectivity and, lead to contrasting dynamics in which hosts show sustained population-level growth. (Parameter values: *r*=2.0, *μ*=0.9, *λ*=0.1, *γ*=0.5, *β*_1_=0.05, *β*_2_=0.1, *μ*_*P*_=0.01, *μ*_*Dm*_=0.01, *r*_*Dm*_=1.5, *K*_*Dm*_=150, *Δ* *T*=*t*/*n*=0.0001)

Defensive microbes affect the interaction and dynamics between the host and the parasite (Fig. 2). In the absence of the defensive microbe, the lagged temporal effect of the parasite (mediated through the link between the within- and between-host processes) leads to periods of growth and decline in host density. Contrasting dynamics occur in the presence of defensive microbes; the dynamical effects of the parasite are reduced and the host dynamics show sustained growth (Fig. 2).

Based on these results, we investigate the determinants of host fitness in the presence of defensive microbes. We begin by defining host fitness (*ω*_*H*_) as:

7 $$ \begin{aligned} \omega_{H}&=\frac{1}{H}\frac{dH}{dt} \\ &=f(r,D_{m})-\lambda(D_{m})\int_{0}^{\infty} \psi(x) P(x) dx-g(\mu,D_{m}) \end{aligned}  $$

This is a Fisherian measure of fitness. If overall net birth rate (*r*−*μ*) decreases as defensive microbes density increases, then the cost function is then assumed to have the simple form:

8 $$ f(r,D_{m})-g(\mu,D_{m})=\frac{r-\mu}{1+D_{m}}.  $$

The change in host fitness due to the defensive microbe is:

9 $$ \frac{\partial\omega_{H}}{\partial D_{m}}=-\frac{(r-\mu)}{(1+D_{m})^{2}}+\frac{\lambda \int_{0}^{\infty} \psi(x) P(x) dx}{(1+D_{m})^{2}}.  $$

The strength of this selection is:

10 $$ \frac{\partial^{2}\omega_{H}}{\partial D_{m}^{2}}=2\frac{(r-u)}{(1+D_{m})^{3}}-2\frac{\lambda \int_{0}^{\infty} \psi(x) P(x) dx}{(1+D_{m})^{3}}.  $$

The fitness differences of harbouring the benefits of defensive microbes for protection against the parasite ($\frac {\lambda \int _{0}^{\infty } \psi (x) P(x) dx}{1+D_{m}}$) versus the costs on fecundity ($\frac {r-\mu }{1+D_{m}}$) are:

11 $$ \begin{aligned} \Delta \omega_{H} &=\overbrace{\left(\frac{r-\mu}{1+D_{m}}-\lambda \int_{0}^{\infty} \psi(x) P(x) dx \right)}^{costs-only} \\ & \qquad -\overbrace{\left(\frac{r-\mu}{1+D_{m}}-\frac{\lambda \int_{0}^{\infty} \psi(x) P(x) dx}{1+D_{m}} \right)}^{costs - benefits}. \end{aligned}  $$

**Fig. 3:**
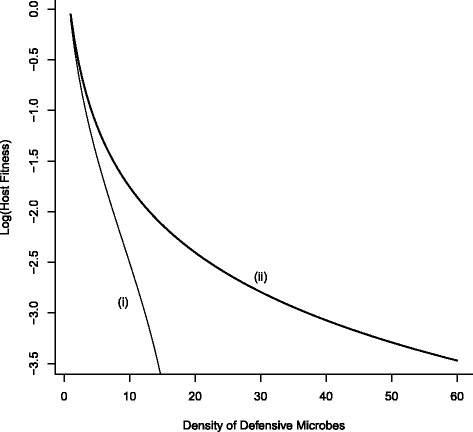
Host fitness as a function of defensive microbe density. Fitness under (i) costs only and (ii) costs and benefits of harbouring defensive microbes. Host fitness decreases as microbe density increases but is lower when benefits of protection from infection accrue. At low microbe density the fitness of the host is of the order $\lambda \int _{0}^{\infty } \psi (x) P(x) dx$ but the fitness difference between costs only and costs-benefits increases (at $\approx (2\lambda \int _{0}^{\infty } \psi (x) P(x) dx)(1+D_{m})^{-3}$). (Parameter values: *r*−*μ*=2.0, *λ*=0.1)

Evaluated when net birth rate is strictly positive (*r*−*μ*>0), at low defensive microbe densities, it is expected that fitness costs will predominate. Hence, it is difficult to discern any selective advantage of low levels of defensive protection. Only as defensive microbe density increases do these differences between costs only (no protection from parasite infections) and full cost-benefits become apparent (Fig. 3).

### Parasite fitness and strength of selection

In this section based on the parasite dynamics within a host (Eq. 1) we evaluate the fitness consequence of defensive microbes on the parasite. Parasite fitness (*ω*_*P*_) is defined as:

12 $$  \omega_{P}=\frac{1}{P}\frac{\Delta P}{\Delta t}=\left[\frac{\gamma}{1+D_{m}}-\beta_{1} D_{m} - \mu\right].  $$

The changes in parasite fitness caused by the defensive microbe can be determined from the first and second partial derivatives of fitness (*ω*_*P*_) with respect to defensive microbe density such that the change in fitness is:

13 $$  \frac{\partial\omega_{P}}{\partial D_{m}}=- \frac{\gamma}{\left(1+D_{m}\right)^{2}}-\beta_{1}  $$

and the strength of selection is:

14 $$  \frac{\partial^{2}\omega_{P}}{\partial D_{m}^{2}}= \frac{2 \gamma }{\left(1+D_{m}\right)^{3}}.  $$

**Fig. 4:**
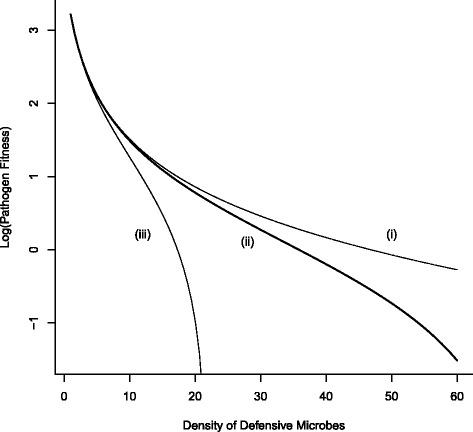
Pathogen fitness as a function of defensive microbe density. Fitness under costs of reduced infectivity and varying strength of interspecific competition (i) *β*_1_=0.001, (ii) *β*_1_=0.01, (iii) *β*_1_=0.1. Competition has an increasing effect on fitness at high defensive microbe density. The only ESS is when the density of defensive microbes is zero. Otherwise fitness declines (Eq. 13) and the strength of selection against the pathogen accelerates (Eq. 14). (Other parameter values: *γ*=50, *μ*_*P*_=0.0)

In general, fitness declines at an accelerating rate as the density of defensive microbes increases. The interplay between reduction in fitness due to the effects of host protection (and lower levels of infectivity) and reductions due to interspecific parasite-defensive microbe competition is a function of microbe density (Fig. 4).

At low densities of defensive microbes, fitness reductions combine both effects due to reduced infectivity and interspecific competition, whereas at high densities, fitness reduction is essentially due to the effects of competition. Furthermore, from Eq. (13) there is no feasible evolutionary stable strategy (ESS) ($\frac {\partial \omega _{P}}{\partial D_{m}} \neq 0$) and the strategy is potentially prone to invasion and replacement by alternative defensive microbe strategies and/or strategies utilizing a different approaches for building immune protection. We explore this further in the next section.

### Evolutionary investment in defensive microbes or immunity

In this section we investigate the evolutionary investment in immunity versus acquiring parasite-protection through defensive microbes. We do this through an invasion analysis by considering that the evolution of an alternative host strategy will establish intense shared parasite-mediated competition. This form of competition between host strategies then defines the ecological trait, the ability to suppress the parasite, that quantifies the interaction between these different host defensive strategies. In making this assumption (rather than a direct interaction simply for resources), we establish the appropriate dynamic game for investigating the evolution of host defenses through microbes or immunity. We explore this dynamic game in three ways; we investigate the invasion conditions, we use numerical simulations and develop an individual-based approach (Additional file 1: Appendix A) to determine the conditions that favour a defensive microbe system over an immunity-based strategy.

As noted, to explore the parasite mediated form of competition and in addition to the host dynamics mediated by defensive microbes, we define an alternative within-host dynamic in which the parasite density is modulated by immunity:

15 $$ \frac{\Delta P}{\Delta T}=\left(\overbrace{\gamma}^{replication}-\overbrace{\mu_{P}}^{death} -\overbrace{\alpha I}^{immunity}\right) P  $$

where *I* is the density of immune cells and, following a mass-action function (e.g., [40, 41]), *α* is the increased rate at which the parasite dies due to the density of immune cells. *γ* is the parasite replication rate and *μ*_*P*_ is the background parasite death rate (as defined in Eq. 1). Within-host immune dynamics are described by:

16 $$ \frac{\Delta I}{\Delta T}=\overbrace{\lambda_{0} P}^{stimulation} -\overbrace{\mu_{I} I}^{death}  $$

where *λ*_0_ is the rate at which immunity is stimulated and *μ*_*I*_ is the loss of immune cells.

Greater protection (and consequently higher host fitness) from defensive microbes is expected to evolve when the (steady-state) parasite density is lower in the presence of these defensive microbes ($P^{*}_{D_{m}}$) than occurs through host immunity ($P^{*}_{I}$) (Additional file 1: Appendix A). That is:

17 $$  P^{*}_{D_{m}}<P^{*}_{I}.  $$

By solving Eqs. 2 and 16 for the steady-state parasite density in the presence of defensive microbes or host immunity, respectively, this inequality (Eq. 17) is:

18 $$  \frac{h(D_{m}^{*})-\mu}{\beta_{2}}<\frac{\mu_{I} I^{*}}{\lambda_{0}}.  $$

The derivation of the inequality (Eq. 17) highlights that simply evaluating parasite loads in the presence of defensive microbes **or** immune-based protection provides a prediction to the outcome of strategy evolution. Explicitly evaluating this inequality (Eq. 17) emphasizes the importance of measuring life history parameters associated with defensive microbe or immune based protection (Eq. 18 – see Additional file 1: Appendix A for further details), and this general finding for the conditions under which defensive microbe protection is favoured is supported by numerical simulation (Additional file 1: Appendix A).

To investigate further these evolutionary invasion dynamics, a defensive microbe based strategy will spread when $\frac {\Delta Dm}{\Delta T}>0$. In the presence of a parasite load set by an immune system ($P=\frac {\mu _{I} I^{*}}{\lambda _{0}}$) a defensive microbe strategy will spread (derived from Eq. 2) when:

19 $$ \frac{h(D_{m})-\mu}{\mu_{I} I^{*}}>\frac{\beta_{2}}{\lambda_{0}}  $$

where *β*_2_ is the rate a which defensive microbes are suppressed by the parasite and *λ*_0_ is the rate at which the immune system is stimulated by the parasite. If the ratio of *β*_2_:*λ*_0_ is large then the defensive microbe strategy will not spread as parasites suppress defensive microbes much more that stimulating the host immune system. Slow decaying immunity (*μ*_*I*_→0) makes the evolution of defensive microbe strategies unlikely as the ratio of *h*(*D*_*m*_)−*μ*:*μ*_*I*_ *I* is likely to be very large. Conversely, rapidly decaying or non-persistent immunity suggests that protection from defensive microbes could be more likely to evolve. These general results on the costs of investment are further corroborated using an individual-based approach (Additional file 1: Appendix A). By explicitly evaluating the individual costs and benefits of investing in different parasite protective strategies, we predict that defensive microbe system could be expected to replace immune-based protection at intermediate costs associated with immunity (Additional file 1: Appendix A).

### Coevolutionary dynamics

In this section, we explore the potential for coevolutionary dynamics on the three species interaction (host, parasite, defensive microbe). As eloquently explained by Slatkin and Maynard Smith [30], models of coevolution have tended to be biased towards genetic interpretations. Yet the ecological context for the different species, interactions and population dynamics is of critical importance to a fuller interpretation of coevolutionary dynamics. Using our phenotypic approach we explore the coevolutionary dynamics of hosts harbouring defensive microbes with parasites by deriving appropriate measures of fitness from Eqs. (1-5). We do this by investigating changes in a *fitness set* to changes in both population densities *and* traits. This *fitness set* is a set of phenotypes for different species characterized by individual measures of fitness [42]. Here, it is the solution to a matrix of partial derivatives (e.g., $\frac {\partial f_{j}(N_{1},\ldots,N_{k})}{\partial N_{j}}$) describing the interactions and is evaluated when the (lead) eigenvalue of this matrix (*θ*) equals zero. This eigenvalue defines the fitness of all the strategies and hence we think of it as a *fitness set*. The fitness set is derived from the solution of the determinant of the following matrix and evaluated when *θ*=0:

20 $$ \left(\begin{array}{ccc} -\theta-\frac{\partial k(H)}{\partial H} & \frac{\partial k(H)}{\partial P} & \frac{\partial k(H)}{\partial D_{m}} \\ \frac{\partial h(P)}{\partial H} & -\theta - \frac{\partial h(P)}{\partial P} & \frac{\partial h(P)}{\partial D_{m}} \\ \frac{\partial j(D_{m})}{\partial H} & \frac{\partial j(D_{m})}{\partial P} & -\theta - \frac{\partial j(D_{m})}{\partial D_{m}} \end{array} \right).  $$

where *k*(*H*) is the host dynamics (from Eq. 5), *h*(*P*) is the parasite dynamics (from Eq. 1) and *j*(*D*_*m*_) is the defensive microbe dynamics (from Eq. 2). More usefully, components of fitness can be investigated by taking the *minors* of this full fitness matrix. In particular, the fitness set associated with the host-parasite interaction is the solution of:

21 $$ Det\left(\begin{array}{cc} -\theta-\frac{\partial k(H)}{\partial H} & \frac{\partial k(H)}{\partial P}\\ \frac{\partial h(P)}{\partial H} & -\theta - \frac{\partial h(P)}{\partial P} \end{array} \right)=0.  $$

Similarly, the fitness set associated with parasites and defensive microbes is the minor:

22 $$ Det\left(\begin{array}{cc} -\theta-\frac{\partial h(P)}{\partial P} & \frac{\partial h(P)}{\partial D_{m}}\\ \frac{\partial j(D_{m})}{\partial P} & -\theta - \frac{\partial j(D_{m})}{\partial D_{m}} \end{array} \right)=0  $$

and finally, the fitness set of hosts and defensive microbes is the minor:

23 $$ Det\left(\begin{array}{cc} -\theta-\frac{\partial k(H)}{\partial H} & \frac{\partial k(H)}{\partial D_{m}}\\ \frac{\partial j(D_{m})}{\partial H} & -\theta - \frac{\partial j(D_{m})}{\partial D_{m}} \end{array} \right)=0.  $$

As noted, coevolutionary dynamics depend on species that have genetic control over traits [30] and we therefore investigate changes in both population densities and traits in each pairwise interaction.

#### Host-parasite minor

Evaluating the direct effects of parasite on host fitness from Eq. 21:

24 $$ \frac{\partial k(H)}{\partial P}=\frac{\partial (\lambda(D_{m}) \int_{0}^{\infty} \psi(x) P(x) dx)}{\partial P} = \lambda(D_{m}) E(P)  $$

highlights the importance that the expected parasite density (*E*(*P*)) has on host fitness. Importantly, this effect is not independent of defensive microbe density as these microbes modulate the infection rate (*λ*(*D*_*m*_)). However, as host density has no direct effect on the dynamics of the parasite ($\frac {\partial h(P)}{\partial H}=0$), the fitness set (**F**) associated with these coevolutionary dynamics is simply the solution to the product of the partial derivatives along the diagonal of the minor matrix (Eq. 21). This turns out to yield the fitness set:

25 $$ \begin{aligned} \mathbf{F} &\ni \left\{f(r,D_{m})-\lambda(D_{m}) \int_{0}^{\infty} \psi(x) P(x) dx\right. \\ & \qquad \left. - g(\mu,D_{m}),\gamma(D_{m})-\beta_{1} D_{m} -\mu {\vphantom{\int_{0}^{\infty}}}\right\} \end{aligned}  $$

**Fig. 5:**
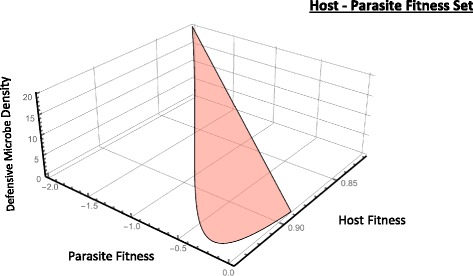
Coevolutionary fitness dynamics for the host-pathogen interaction. Admissible fitness set based on Eq. 25 illustrates the role of defensive microbe density on both host and parasite fitness. Increasing defensive microbes reduces parasite fitness (through reducing infectivity) and host fitness (through effects on net population growth). (Default parameter values *r*=2.0, *μ*=0.01, *λ*=0.01,*γ*=1.1, *β*_1_=0.01, *β*_2_=0.01, *μ*_*P*_=0.01, *μ*_*Dm*_=0.01, *r*_*Dm*_=4.0, *K*_*Dm*_=10)

This fitness expression reveals that the interaction between the host and parasite is mediated, indirectly, through defensive microbes affecting the cost of host reproduction and infection in the host ($f(r,D_{m})-\lambda (D_{m}) \int _{0}^{\infty } \psi (x) P(x) dx -g(\mu,D_{m})$) and, parasite replication and competition (*γ*(*D*_*m*_)−*β*_1_ *D*_*m*_−*μ*). Figure 5 illustrates the admissible fitness set for the host-parasite coevolutionary dynamics. At low defensive microbe densities, host and parasite fitness is positive but increases in defensive microbe densities, exert increasing costs on both parasite infectivity and host population growth. Beyond critical levels of defensive microbe densities, coevolution of host-parasite interaction is no longer feasible as (i) the costs of harbouring defensive microbes are greater than the benefits that they provide and (ii) the increased competitive effects of defensive microbe competition has the potential to reduce parasite infectivity.

#### Parasite-defensive microbe minor

**Fig. 6:**
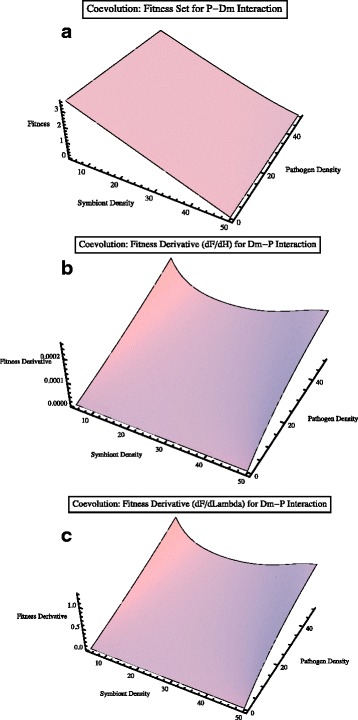
Coevolutionary fitness dynamics for the pathogen-defensive microbe interaction. **a** The fitness set of defensive microbes - pathogen interaction is predominantly determined by the density of defensive microbes. As defensive microbes increase in density, coevolutionary fitness (associated with the interaction) weakens as defensive microbes (i) increase competitive effects on pathogens and (ii) reduce pathogen infectivity. **b** Fitness derivative (*dF*/*dH*) due to hosts on fitness set (coevolution) of defensive microbes and pathogens. **c** Fitness derivative with respect to infection rate (*λ*) (in the defensive microbe-pathogen fitness set). Fitness changes are positive when the density of defensive microbes is very low and pathogen density is high. As defensive microbes increase in density, this reduces infection potential and increases the effects of competition with the pathogen so the changes in selection declines. When pathogens are at low density, this change in selection is weakened. (Default parameter values *r*=2.0, *μ*=0.01, *λ*=0.01,*γ*=1.1, *β*_1_=0.01, *β*_2_=0.01, *μ*_*P*_=0.01, *μ*_*Dm*_=0.01, *r*_*Dm*_=4.0, *K*_*Dm*_=100)

The fitness set and hence the coevolutionary dynamics of the defensive microbe-parasite interaction is predominantly determined by the density of the defensive microbes (Fig. 6 a-c). As defensive microbes increase in density, the fitness set associated with the interaction weakens (**F**→0) given that the defensive microbes both increase competitive effects on parasites and reduce parasite infectivity.

Changes in the effects of parasite infectivity on the fitness of the parasite-defensive microbe interaction have a range of outcomes (Fig. 6 c). When parasites are rare, the change in selection is weakened so there is little fitness response ($\frac {d\mathbf {F}}{d\lambda } \to 0$). However, when the density of defensive microbes is low and parasite density high, there is a strong positive effect of changes in infectivity rate on fitness set of the interaction (as the parasite is essentially unaffected by the inhibitory effects of the defensive microbe). As the density of defensive microbes increases, these microbes further reduce the infection potential and the strength of selection on the parasite-defensive microbe interaction (Fig. 6 c).

#### Host-defensive microbe minor

As host density has no direct effects on the dynamics of the defensive microbes (Eq. 2), the fitness set (**F**) associated with the host and defensive microbe interaction is simply the fitness on the diagonal of the minor matrix associated with the independent host ($\frac {\partial k(H)}{\partial H}$) and defensive microbe ($\frac {\partial j(Dm)}{\partial Dm}$) dynamics (Eq. 23):

26 $$ {}\mathbf{F} \ni \left\{\frac{r-u-\lambda \int_{0}^{\infty} \psi(x) P(x) dx }{1+D_{m}},-\beta_{2} P-\mu+h^{\prime}(D_{m})\right\}  $$

where *h* ^′^(*D*_*m*_) is the derivative of the growth rate of the defensive microbes in the host (see Eq. 2). The host-defensive microbe coevolutionary dynamics are then essentially mediated through the parasite which has negative effects on hosts (through infection: $\lambda \int _{0}^{\infty } \psi (x) P(x) dx$) and defensive microbes (through competition: *β*_2_ *P*).

## Discussion

The role of microbes in protecting hosts from infection has been observed in a diversity of animal and plant species [3, 5–9]. Defensive microbes have been shown to ‘take-over’ from or complement the host’s own defences, driving variation in resistance and underlying host-parasite specificity [25, 28, 43]. Here, we have used a theoretical framework to show that these microbes can spread throughout a host population and engage in tripartite coevolution with hosts and parasites.

We show that the outcome of the evolutionary dynamics of defensive microbes can be highly dependent on the costs and benefits associated with microbe-mediated versus host-encoded resistance to parasites. In nature, defensive microbes demonstrate a profound ability to spread [7, 23, 44]. The spread of *Spiroplasma*, a defensive symbiont in *Drosophila neotestacea* throughout North America was recently captured and hypothesised to be driven by selection from sterilizing nematode parasites [7]. Hosts may not maintain or evolve their own resistance or immunity given the costs of immune system activation, maintenance and evolution [45] as defensive microbes might be cheaper. This cost imbalance could lead to the redundancy in some or all of host immune function, a finding consistent with studies showing that hosts with an evolutionary history of possessing defensive microbes can lack major immune system components [46, 47]. Recent work has revealed that microbe-mediated protection can even reduce selection for host-based resistance to parasites [48]. This response, however, does not include protective mechanisms involving modulation of the immune system [49, 50]. The model presented, however, provides a framework for examining such issues.

Given the impact of defensive microbes on host and parasite fitness, it has been speculated that they are likely to influence host-parasite coevolution [51, 52]. We have explicitly included defensive microbes as a third species in our model as a tripartite set of coevolutionary interactions in which hosts do not possess an immune response to the parasites, but can only defend themselves using microbes. Our tripartite model highlights the interplay of two types of antagonism on coevolution - defensive microbe-parasite competition and host-parasite interactions. Partitioning the coevolutionary dynamics yields testable predictions. We find that the strength of selection resulting from host-parasite coevolution is directly affected by defensive microbes more so at higher microbe densities when the costs to the host outweigh the benefits afforded from protection. Coevolutionary dynamics between defensive microbes and parasites are also predominately driven by changes in microbe density which correspondingly alter competitive effects and parasite infectivity. While the mechanisms of microbe-mediated protection observed in nature are remarkably diverse [12], principally, defensive microbes and parasites have been observed to act antagonistically [53]. The positive (for the host) and negative (for the parasite) outcomes associated with competitive interactions play a key role in the coevolutionary dynamics explored here (Fig. 3).

The within-host density of defensive microbes likely plays a key role in determining the outcome of host-defensive microbe-parasite interactions. Understanding the relationship between within-host defensive microbe density and the strength of protection conferred, particularly if parasite suppression occurs via resource competition or toxin production (i.e., more bacterial cells increase total toxin concentration) [24], is critical in determining potential coevolutionary outcomes. In our model, costs are intimately linked to density. Consequently, we find that intermediate levels of defensive microbe densities are ideal for facilitating host-parasite coevolution. The costs and benefits of these relationships remain to be explored by directly manipulating within-host densities in defensive symbioses. Such experimental manipulations would be valuable as studies (e.g., [54–56]) have shown that other factors can confound the links between density and the strength of defensive microbe protection (e.g., host age) and associated costs to the host (e.g., genotype by genotype interactions, superinfection).

Variability in parasite infection rates is another aspect of our model that requires empirical validation. Coevolution between the host and defensive microbe is an indirect effect mediated through the parasite that has negative impacts on the hosts (through infection) and defensive microbes (through competition). When parasite densities are low, defensive microbes negatively affect host fitness due to costs. However, at high parasite densities, defensive microbes can cross the parasite-mutualist continuum and offset their costs by competing with coevolving parasites. Model predictions make it clear that parasites must be abundant for defensive microbes to benefit hosts enough to overcome the costs (Fig. 5), a finding consistent with previous studies [24, 57, 58]. Parasite pressure is not always constant however, and it is likely to be spatially variable [59, 60] or have a natural periodicity (e.g., induced by seasonality [61]. Although explored only peripherally in theory [62, 63] and once directly tested [44], the extent to which this variation alters the intensity of selection for defensive microbes is important for predicting their spread and evolutionary impact.

## Conclusions

The presence of defensive microbes in a diversity of host-parasite systems challenges our fundamental understanding of the ecology and evolution of infectious disease. Our results provide theoretical insights into the coevolutionary implications of microbe-mediated protection to host-parasite interactions. In summary, our model suggests that a parasite-defence strategy involving the acquisition of defensive microbes demonstrates an intense ability to spread in host populations. We show that defensive microbes compete with coevolving parasites when at high densities, ultimately reducing parasite infectivity and protecting the hosts. Microbes can therefore occupy a central role in host-parasite interactions on an evolutionary timescale, with the potential to drive tripartite coevolution.

## Additional file

Additional file 1 Appendix A. In this supplementary material we present the mathematical details for the evolutionary investment in defensive microbe or immune based systems. Together with the details of the invasion analysis mediated by intense shared parasitism, these results on the evolution of a defensive microbe system are corroborated with numerical simulations and an individual-based approach. (PDF 3130 kb)
