# Supplementary material for: The evolutionary and coevolutionary consequences of defensive microbes for host-parasite interactions
Source: BMC Evol Biol. 2017 Aug 14;17:190. doi: 10.1186/s12862-017-1030-z (PMC5557575; doi:10.1186/s12862-017-1030-z)
Supplement: Supplementary file 1 — Appendix A. In this supplementary material we present the mathematical details for the evolutionary investment in defensive microbe or immune based systems. Together with the details of the invasion analysis mediated by intense shared parasitism, these results on the evolution of a defensive microbe system are corroborated with numerical simulations and an individual-based approach. (PDF 3130 kb) [file 12862_2017_1030_MOESM1_ESM.pdf]

Appendix A

Evolutionary Investment in Defensive Microbes or Immunity

In this appendix we outline the derivation of inequality condition (eqn 17) that greater protection and consequently higher host fitness occurs when (steady state) parasite density is lower in the presence of defensive microbe than occurs through host immunity:

$$P^*_{D_m} < P^*_I.$$

To investigate the evolution of investment in immunity versus acquiring protection through defensive microbes, we define an alternative host strategy that acquires protection through immunity:

$$\frac{dH_I}{dt} = \left[f(r, I) - \lambda(I) \int_0^{\infty} \psi(x) P_I(x) dx - g(\mu, I) \right] H_I$$

where the host dynamics (H_I) are governed by costs and benefits of investment in immunity (to level I). $f(r, I)$ and $g(\mu, I)$ are the birth rate and death rate of hosts, respectively, under costly investment in immunity. $\lambda(I)$ is the clearance of parasites P_I by immune cells. More explicitly, costly investment in immunity by hosts can be described by:

$$\frac{dH_I}{dt} = \left[\frac{r - \mu}{1 + I} - \lambda_I \int_0^{\infty} \psi(x) P_I(x) dx \right] H_I.$$

where λ_I is the parasite clearance rate by immune cells. Taking net growth rate ($\frac{1}{H_I}\frac{dH_I}{dt}$) as a measure of (Fisherian) fitness, hosts acquiring protection through immunity have fitness:

$$\frac{1}{H_I}\frac{dH_I}{dt} = \frac{r - \mu}{1 + I} - \lambda_I \int_0^{\infty} \psi(x) P_I(x) dx.$$

Host dynamics where defensive microbes provide protection from parasites (eqn 7) is defined as:

$$\frac{dH}{dt} = \left[f(r, D_m) - \lambda(D_m)\int_0^\infty \psi(x)P_{D_m}(x)dx - g(\mu, D_m)\right]H$$

where $f(r, D_m)$ and $g(\mu, D_m)$ are birth rate and death rate of hosts, respectively, under costly investment in defensive microbes (D_m), and $\lambda(D_m)$ is the clearance of parasites P_{D_m} by defensive microbes. More explicitly:

$$\frac{dH}{dt} = \left[\frac{r-\mu}{1+D_m} - \frac{\lambda_{max}\int_0^\infty \psi(x)P_{D_m}(x)dx}{1+D_m}\right]H.$$

Host fitness in the presence of the defensive microbes is then:

$$\frac{1}{H}\frac{dH}{dt} = \frac{r-\mu}{1+D_m} - \frac{\lambda_{max}\int_0^\infty \psi(x)P_{D_m}(x)dx}{1+D_m}.$$

For a defensive microbe system to evolve then host fitness in the presence of defensive microbe must be greater than the fitness in the presence of immune system protection:

$$\frac{1}{H}\frac{dH}{dt} > \frac{1}{H_I}\frac{dH_I}{dt}$$

which gives:

$$\frac{r-\mu}{1+Dm} - \frac{\lambda_{max}\int_0^\infty \psi(x)P_{Dm}(x)dx}{1+Dm} > \frac{r-\mu}{1+I} - \lambda_I\int_0^\infty \psi(x)P_I(x)dx.$$

Under no costs ($\frac{r-\mu}{1+D_m} \equiv \frac{r-\mu}{1+I}$, $\frac{1}{1+D_m} \to 1$) and equivalent clearance rates $\lambda_{max} \equiv \lambda_I$ then this inequality simplifies to:

$$P^*_{D_m} < P^*_I.$$

Defensive microbe protection is expected to evolve when it leads to lower parasite densities than occurs when host immunity provides protection.

Numerical Simulation

To illustrate that the inequality condition ($P^*_{D_m} < P^*_I$) holds we simulate the parasite (P) dynamics in the presence of defensive microbes ((D_m) or host immunity (I). Under defensive microbe protection, the within-host dynamics are governed by:

$$\frac{dP}{dt} = (\gamma - \mu_P - \alpha_{Dm} D_m)\, P$$

$$\frac{dD_m}{dt} = \left(-\beta_2 P - \mu_{Dm} + r\left(1 - \frac{D_m}{K_{Dm}}\right)\right)$$

where γ is the parasite replication rate, μ_P is the background pathogen death rate and α_{Dm} is the increased rate at which parasites die due to the effects of the defensive microbes.

Under host immunity, the within-host dynamics are governed by:

$$\frac{dP}{dt} = (\gamma - \mu_P - \alpha_I I)\, P$$

$$\frac{dI}{dt} = \lambda_0 P - \mu_I I$$

where α_I is the increased rate at which parasites die due to the effects of the immune system, λ_0 is the rate at which the immune system is stimulated and μ_I is the loss of immune cells. Other parameters are as defined above.

Figure 1 shows the outcome of a numerical simulation of these dynamics. In the presence of defensive microbes, parasites are eliminated ($P^*_{Dm} = 0$) whereas in the presence of host immunity, parasite steady state density is greater ($P^*_I \approx 11.75$). Host fitness in the presence of defensive microbes is then expected to be higher than that achieved under immunity. Variation in the costs of immunity or protection through defensive microbes will influence whether a

defensive microbe strategy will spread and persist. Costly host investment in immunity and/or defensive microbes will modify the inequality and depending on the relative magnitude of these costs determine when a defensive microbe protection will evolve.

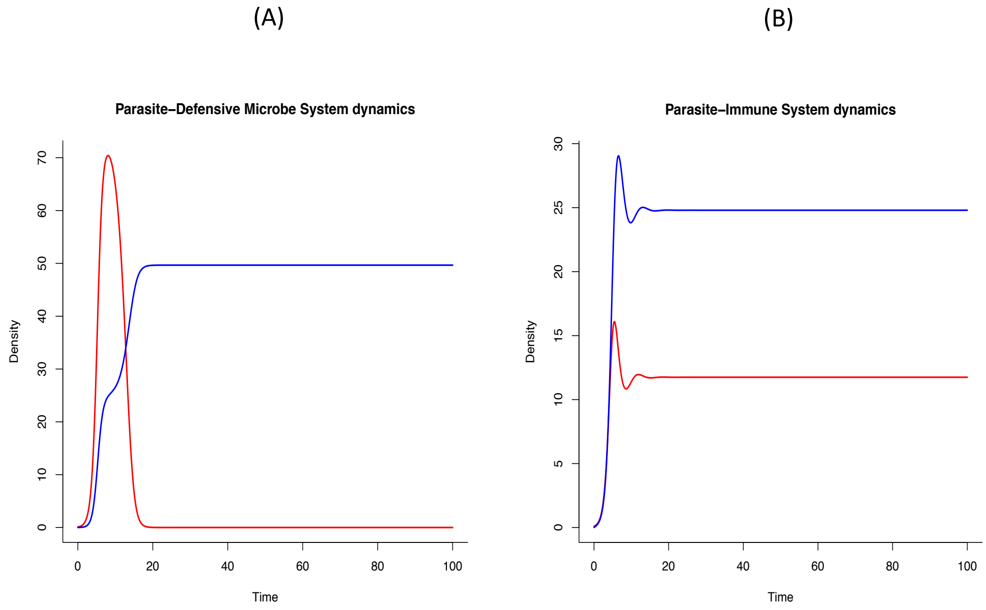

Figure 1: Numerical dynamics of parasite dynamics (red line) under (a) defensive microbe (blue line) system and (b) host immune (blue line) system. Defensive microbe systems lead to lower pathogen levels than immune-based systems and as such are expected to give rise to higher host fitness. Differential equations solved using a Runge-Kutta (4th order) method with a fixed parameters set [$\gamma = 1.25$, $\mu_P = 0.01$, $\alpha_I = \alpha_{Dm} = 0.05$, $\lambda_0 = 1.9$, $\mu_I = 0.9$, $\mu_{Dm} = 0.01$, $r = 1.5$, $K = 50$, $\beta_2 = 0.01$] and initial conditions: $P(0) = 0.01$, $D_m(0) = 0.01$ and $I(0) = 0.01$.

Individual-based approach to the evolution of defensive microbe system

To compliment the population-level approaches (invasion analysis, numerical simulations), in this section we outline an individual-based approach to understand when a defensive microbe system strategy would be evolutionarily more likely than an immunity-based strategy.

Assumptions

In this individual-based approach for the evolution of defensive microbe protection we make two main assumptions:

- A host chooses to invest in protection from **either** defensive microbes or innate immunity.
- A host gives birth with probability Pr(birth), becomes infected with probability Pr(infected) or dies with probability Pr(death). These probabilities are dependent on whether a host has innate immunity or defensive microbe protection.

These simple assumptions for the individual-based model set up a system which can be in one of four states: hosts with innate immunity (H_I), host with defensive microbe (H_{Dm}), infected host (P) and empty (0).

The infected host (P) and empty (0) states are essentially hidden. We focus on whether we directly observe a H_I or H_{Dm} state. Once a host is infected it enters an infected host (P) state - in the next time step, the infected host state moves to empty (0). Empty states can only be colonized by hosts.

Transition Matrix

This four state system can be represented by a Markov transition matrix:

$$\mathbf{S}_{t \text{ to } t+1} = \begin{array}{c} \\ H_I \\ H_D \\ P \\ 0 \end{array} \begin{array}{cccc} H_I & H_D & P & 0 \\ \end{array}$$

$$\mathbf{S}_{t \text{ to } t+1} = \begin{array}{c|cccc} & H_I & H_D & P & 0 \\ \hline H_I & Pr(H_I \text{ birth}) & 0 & Pr(H_I \text{ infection}) & Pr(H_I \text{ death}) \\ H_D & 0 & Pr(H_D \text{ birth}) & Pr(H_D \text{ infection}) & Pr(H_D \text{ death}) \\ P & 0 & 0 & 0 & Pr(P \text{ death}) \\ 0 & Pr(H_I \text{ birth}) & Pr(H_D \text{ birth}) & 0 & 0 \end{array}$$

Using methods from linear algebra, we can determine the long-term outcome of the individual-based approach from this Markov transition matrix. A linear sytem ($\mathbf{S}$) can be decomposed into eigenvalues and eigenvectors using $\text{Det}\,(\lambda I - \mathbf{S})$ to give:

$$\mathbf{SN} = \mathbf{\Lambda N}$$

where $\mathbf{\Lambda}$ is a diagonal matrix of eigenvalues and $\mathbf{N}$ is square matrix of eigenvectors of $\mathbf{S}$. So

$$\mathbf{S} = \mathbf{N\Lambda N}^{-1}.$$

Then for the long-term outcome, the state $\mathbf{S}$ in the next time point is:

$$\begin{aligned} \mathbf{S}^2 &= \left(\mathbf{N\Lambda N}^{-1}\right)\left(\mathbf{N\Lambda N}^{-1}\right) \\ \mathbf{S}^2 &= \mathbf{N\Lambda}^2\mathbf{N}^{-1}. \end{aligned}$$

So

$$\mathbf{S}^n = \mathbf{N\Lambda}^n\mathbf{N}^{-1}.$$

We can use this spectral (eigen)decomposition of a Markov matrix to investigate the long-term probability of investing in a defensive microbe system over innate immunity. The probabilities

of host birth, death or infection establish parameter space to explore different probabilistic outcomes for a defensive microbe system as parameter values change.

Defensive Microbe System

For the defensive microbe system, we define the following individual transitions. As we are considering individuals and the contact between individual hosts and pathogens we can represent the costs and benefits of different protections as constants.

As such, the host birth rate is $\frac{r}{1+\alpha}$, the host death rate is $\frac{\mu}{1+\alpha}$ and the host infection rate is $\frac{\lambda}{1+\beta}$, where α and β are the costs and benefits of choosing a particular protection. The death rate of the pathogen is μ_P.

The resulting Markov transition matrix is then:

$$\mathbf{S}_{t \text{ to } t+1} = \begin{array}{c} \\ H_I \\ H_D \\ P \\ 0 \end{array} \begin{array}{c} \begin{array}{cccc} H_I & H_D & P & 0 \end{array} \\ \left[\begin{array}{cccc} \frac{r_I}{N(1+\alpha_I)} & 0 & \frac{\lambda_I}{N} & \frac{\mu_I}{N(1+\alpha_I)} \\ 0 & \frac{r_D}{M(1+\alpha_D)} & \frac{\lambda_D}{M(1+\beta_D)} & \frac{\mu_D}{M(1+\alpha_D)} \\ 0 & 0 & 0 & 1 \\ \frac{r_I}{L(1+\alpha_I)} & \frac{r_D}{L(1+\alpha_D)} & 0 & 0 \end{array} \right] \end{array}$$

where N, M and L are normalisation constants to ensure transitions are probabilities that across each row sum to 1. The benefits of having a defensive microbe system compared to innate immunity are expressed in terms of the probability of infectivity. For hosts with innate immunity this is $\frac{\lambda_I}{N}$ while for hosts with defensive microbes this is $\frac{\lambda_D}{M(1+\beta_D)}$; we scale pathogen infectivity by the benefits provided by defensive microbes. As the infected host state always gives an empty state in the next time step, the probability of infected host mortality is 1.

With this individual-based approach, we can undertake a (stochastic) sensitivity analysis of the deterministic model approaches (invasion analysis; numerical simulations). Figure A2 shows the probability of investing in the defensive microbe system as a function of the costs of immunity (α_I) and the benefits of defensive microbes (β_D) As costs of immunity and benefits of defensive microbes increase, defensive microbe systems are favoured over immunity-based strategies. However, this defensive microbe system can be expected at **intermediate** levels of costs of immunity (Pr(defensive microbes) > 0.5 when $\alpha_I > 0.6$) and benefits of defensive microbes Pr(defensive microbes) > 0.5 when $\beta_D > 0.6$).

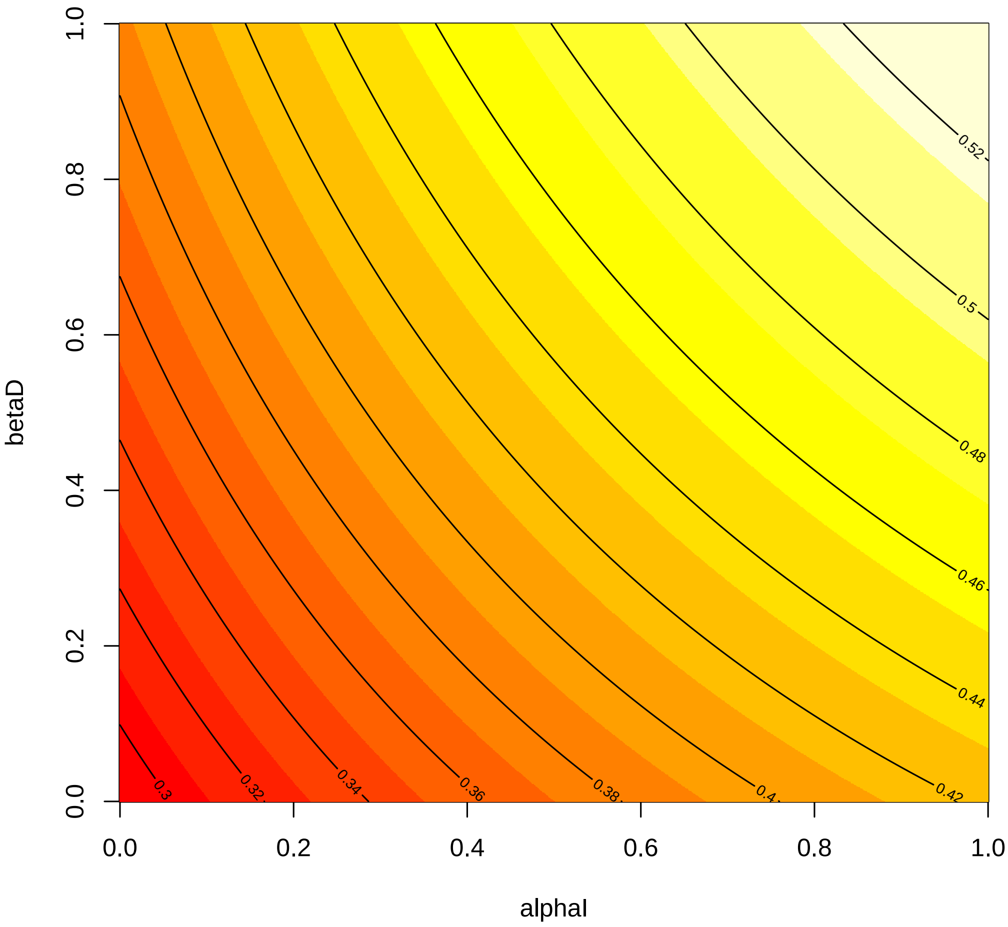

Figure 2: Probability of defensive microbe system (over immunity-based strategies) for costs of immunity (α_I) and benefits of microbes (β_D). Contours show points of equal probability and lighter colours (yellow) higher probability of defensive microbe systems. Individual-based approach confirms that defensive microbe system can spread when immunity is costly [Other parameters: $r_I = r_D = 2.0$, $\lambda_I = \lambda_D = 0.8$, $\alpha_{Dm} = 0.1$, $\mu_I = \mu_{Dm} = 0.1$].
